# Commentary: Accuracy of Clinical Examination in Suspected Pediatric Scaphoid Fractures—A Systematic Review

**DOI:** 10.1177/22925503261460023

**Published:** 2026-06-18

**Authors:** Aneesh Karir, Sydnee Tuckett

**Affiliations:** 1 Division of Plastic and Reconstructive Surgery, Department of Surgery, 8664 University of Manitoba, Winnipeg, Manitoba, Canada

Pediatric hand and wrist injuries remain a common yet diagnostically challenging presentation, often requiring clinicians to balance the risks of missed injury against the consequences of overtreatment. Lamanna et al address an important and underexplored challenge in pediatric hand trauma: the diagnosis of occult scaphoid fractures using clinical examination alone.^1^

Despite scaphoid fractures being the most common carpal fracture in children,^2^ clinicians continue to rely heavily on empiric immobilization and serial imaging because reliable pediatric-specific clinical decision tools remain lacking. The clinical implications of this diagnostic uncertainty are significant. Missing a true scaphoid fracture risks nonunion, avascular necrosis, and possible carpal collapse, while overdiagnosis results in unnecessary immobilization and increased healthcare costs.^3,4^

A major strength of this systematic review is its synthesis of a sparse and heterogeneous body of literature while identifying important gaps in current evidence. Across the five included studies, anatomic snuffbox tenderness emerged as the most commonly assessed clinical finding. Although highly sensitive in one study (100%), its extremely poor specificity (8%) reinforces the limitations of relying on isolated examination findings in clinical practice.^4^ Additional maneuvers, including pain with radial deviation and axial thumb loading, demonstrated variable diagnostic utility, but no individual test consistently demonstrated sufficient accuracy to independently guide management decisions.

The findings of Lamanna et al parallel broader efforts within pediatric hand surgery to develop evidence-based clinical prediction tools. Hartley et al, in their external validation of the Calgary Kids’ Hand Rule (CKHR), demonstrated that combining examination findings with radiographic characteristics can achieve high sensitivity for identifying clinically significant pediatric hand fractures.^5^ The CKHR achieved sensitivities of 91.1% and 98.3% across two tertiary pediatric centers, supporting the concept that structured multivariable prediction models may safely guide triage and referral pathways. Although the CKHR focuses only on phalangeal and metacarpal fractures rather than scaphoid injuries, it provides an important proof-of-concept for pediatric hand trauma care.

In contrast, no validated pediatric-specific clinical prediction rule currently exists for suspected scaphoid fractures. This likely contributes to the continued reliance on defensive immobilization and repeat imaging pathways. The low specificity of individual examination maneuvers shown in this review suggests that future progress may require multivariable approaches integrating clinical findings, patient characteristics, and imaging features rather than isolated physical examination tests alone.

Importantly, the limitations of this review largely reflect deficiencies in the existing literature rather than flaws in methodology. Significant heterogeneity in study design, examination techniques, imaging reference standards, and follow-up intervals precluded meaningful meta-analysis.

Ultimately, Lamanna et al provide an important contribution by emphasizing the need for prospective pediatric studies aimed at developing validated scaphoid-specific prediction rules. Such advances may reduce unnecessary immobilization and healthcare utilization while maintaining patient safety in this diagnostically challenging population.
